# Teleost Piscidins—In Silico Perspective of Natural Peptide Antibiotics from Marine Sources

**DOI:** 10.3390/antibiotics12050855

**Published:** 2023-05-05

**Authors:** Patricia Asensio-Calavia, Sergio González-Acosta, Andrea Otazo-Pérez, Manuel R. López, Antonio Morales-delaNuez, José Manuel Pérez de la Lastra

**Affiliations:** 1 Biotechnology of Macromolecules Research Group, Instituto de Productos Naturales y Agrobiología (IPNA-CSIC), Avda. Astrofísico Francisco Sánchez, 3, 38206 San Cristóbal de La Laguna, Spain; 2 School of Doctoral and Graduate Studies, Universidad de La Laguna, Avda. Astrofísico Francisco Sánchez, SN. Edificio Calabaza-Apdo. 456, 38200 San Cristóbal de La Laguna, Spain

**Keywords:** antimicrobial peptide, fish, piscidin, Teleost, innate immunity, docking, in silico, immunomodulation

## Abstract

Fish, like all other animals, are exposed to constant contact with microbes, both on their skin and on the surfaces of their respiratory and digestive systems. Fish have a system of non-specific immune responses that provides them with initial protection against infection and allows them to survive under normal conditions despite the presence of these potential invaders. However, fish are less protected against invading diseases than other marine vertebrates because their epidermal surface, composed primarily of living cells, lacks the keratinized skin that serves as an efficient natural barrier in other marine vertebrates. Antimicrobial peptides (AMPs) are one type of innate immune protection present in all life forms. AMPs have been shown to have a broader range of biological effects than conventional antibiotics, including antibacterial, antiviral, antiprotozoal, and antifungal effects. Although other AMPs, such as defensins and hepcidins, are found in all vertebrates and are relatively well conserved, piscidins are found exclusively in Teleost fish and are not found in any other animal. Therefore, there is less information on the expression and bioactivity of piscidins than on other AMPs. Piscidins are highly effective against Gram-positive and Gram-negative bacteria that cause disease in fish and humans and have the potential to be used as pharmacological anti-infectives in biomedicine and aquaculture. To better understand the potential benefits and limitations of using these peptides as therapeutic agents, we are conducting a comprehensive study of the Teleost piscidins included in the “reviewed” category of the UniProt database using bioinformatics tools. They all have amphipathic alpha-helical structures. The amphipathic architecture of piscidin peptides and positively charged residues influence their antibacterial activity. These alpha-helices are intriguing antimicrobial drugs due to their stability in high-salt and metal environments. New treatments for multidrug-resistant bacteria, cancer, and inflammation may be inspired by piscidin peptides.

## 1. Introduction

There is growing evidence that the equilibrium of all living things, including those that live in water, depends on a constant dialog with the microbes that cover their surfaces [1]. The diversity of microorganisms in the oceans is probably underestimated. If a subset of these bacteria is indeed harmful to marine fishes, it is plausible that coevolutionary processes drove the evolution of innate antimicrobial defenses in these organisms. Consequently, it is reasonable to expect a corresponding set of such defenses in fish [2]. Fish express all major AMP families, including defensins, cathelicidins, hepcidins, and histone peptides [3]. Bony fishes are divided into two subclasses: Holostei and Teleostei, with the latter being the most important and abundant, comprising 96% of all fish species [4]. Holostei, such as North American bowfins (Amiiformes) and gars (Semionotiformes), are the closest living relatives of Teleostei. Teleosts inhabit various niches and are at increased risk of being attacked by a variety of potential pathogens [5]. The main structural difference between Teleostei and other bony fishes is in the jaw bones. Teleostei have a movable premaxilla and accompanying changes in jaw musculature that allow them to extend their jaws out of their mouths. This gives them a significant advantage, allowing them to grab their prey and guide it into their mouth. They also have similar-sized caudal fin lobes and a spine that ends at the caudal peduncle [6,7]. Under normal circumstances, fish can cope with these potential invaders thanks to a system of non-specific immune responses that provides an initial defense against pathogens. Several pathogens enter fish because their skin, gills, and intestines are the main body surfaces that come into direct contact with the environment [8]. However, the exact methods by which hosts interact with their microbiome and the ways in which the emergence of a species’ microbiome is regulated are still largely unclear [9].

The Teleost skin consists of two main parts: the epidermis and the dermis. The cells on the surface are not keratinized [10]. When the differentiation process of the mucous cells begins in the stratum germinativum, the nascent cells migrate to the skin surface and secrete their contents [11]. Fish do not have lymph nodes or other lymphatic tissue as found in mammals [12]. Most pathogens enter Teleosts through the mucous membranes, which are the main point of contact between fish and their immediate environment [13]. The cells that produce the alarm substance and melanophores are located in the dermis or deeper epidermis and do not reach the skin surface. Injury to the epidermis is the only trigger for the release of the alarm substance stored in the skin cells. Both the epidermis and dermis are necessary for skin health [14].

Although the skin, gills, and intestinal mucosa are constantly exposed to microorganisms from the environment, infections or life-threatening lesions do not occur under normal conditions [15]. Antimicrobial peptides (AMPs) are one type of innate immune protection [16,17]. In vertebrates, low-molecular-weight antibacterial peptides are typically found in peripheral blood leukocytes and on mucous membranes [8]. Similarly, mucus extracts from the skin of several fish species, including rainbow trout, have been identified as having antimicrobial peptides against selected bacteria [18]. Host defense peptides have been found to have close genetic, structural, and functional relatives in non-fish species thanks to advances in genome sequencing research [19,20]. Recent research has shown that the peptide piscidin is synthesized in the epithelia of the gills, skin, stomach, and gut of a variety of Teleost species. Piscidins are present in eosinophil cells in epithelial tissues, suggesting that they play an important role in innate defense in these tissues [21].

Silphalin and Noga discovered the first piscidin in the hybrid striped bass (*Morone saxatilis* × *Morone chrysops*) [22]. Piscidins are a family of cationic AMPs that are produced by fish and have broad-spectrum antimicrobial activity against bacteria, fungi, and viruses [23]. The piscidin family includes the well-studied peptides Epinecidin-1, Myxinidin, Chrysophin, Diacentracin, Pleurocidins, and Moronecidins, all of which play important roles in innate immunity in fish [23,24]. They are relatively short peptides, typically consisting of 22–40 amino acids, and are rich in arginine and cysteine residues [25]. Piscidins are primarily produced by fish leukocytes, including mast cells, neutrophils, and macrophages, and are stored in granules within these cells [26]. They are released in response to microbial infections or other inflammatory stimuli and act by disrupting the membranes [27]. Using immunohistochemistry, researchers identified piscidins in the tissues of fishes of the families Moronidae, Sciaenidae, Serranidae, Cichlidae, Siganidae, and Belontidae [21]. 

Because of their broad-spectrum antimicrobial activity and immunomodulatory effects, piscidins could be useful in treating a variety of infectious and inflammatory diseases. Researchers are also investigating the potential use of piscidins as a therapeutic agent in humans [28]. One of the most remarkable features of piscidins as potential therapeutic agents is their broad-spectrum antimicrobial activity. Studies have demonstrated the efficacy of piscidins against a wide range of microorganisms, including bacteria, fungi, and viruses, which makes them potentially useful for treating a variety of infectious diseases [29]. Another notable feature of piscidins is their ability to modulate the immune response. Piscidins have been shown to stimulate the production of cytokines and chemokines, which can help to recruit immune cells to sites of infection or injury. They can also promote wound healing and have anti-inflammatory effects [30,31]. Piscidins are relatively small peptides, which makes them easier to synthesize than larger proteins. This could make them a more cost-effective and accessible therapeutic option than other antimicrobial agents [32,33]. Resistance to antibiotics caused by their overuse has been a concern for some time. Many bacterial infections are becoming more common and widespread, with devastating effects on human health [34,35]. With their potent antimicrobial activity and distinct antimicrobial processes, piscidins could be a good substitute for current antimicrobials and offer an advantage over conventional antibiotics in the fight against drug-resistant bacterial diseases [36]. Because piscidins are naturally produced by fish, they may be less likely to cause adverse effects in humans than synthetic antimicrobial agents [37]. However, more research is needed to fully understand the potential benefits and limitations of using piscidins in humans [38].

Marine organisms release complex and diverse chemical compounds to promote their competitiveness and survival. These metabolites have numerous applications, including industry, medicine, drug delivery, and nanotechnology [39]. Earlier methods for studying host defense peptides in fish were time consuming and costly. They required the collection of a large biomass sample, the homogenization of the sample, the extraction and purification of the peptides in a series of multidimensional protein purification steps, and subsequent testing of their activity [2]. The unique combination of marine biotechnology and nanotechnology has made marine bio-nanotechnology one of the most promising biotechnologies and scientific research fields of study today [39]. The breakthrough came about because genomic screening was used in place of time- and labor-intensive traditional screening methods [40]. Using this method, new pleurocidins were discovered from winter flounder [41]. However, one of the difficulties of the study of piscidin proteins is concerned with the processing and maturation of the active peptide when it is separated from the pro-peptide. There are bioinformatic tools for the prediction of the signal peptide cleavage site, such as Signal-P (https://services.healthtech.dtu.dk/services/SignalP-6.0, accessed on 14 March 2023), which are really helpful [42]. Although several studies have been conducted on the expression and bioactivity of piscidin activity in numerous fish species, only a dozen out of 360 sequences have been reviewed and define the mature active peptide. To better understand the potential benefits and limitations of using these peptides as therapeutic agents, we use bioinformatics methods to perform a comprehensive review of the Teleost piscidins found in the “reviewed” category of the UniProt database (accessed on 14 March 2023).

## 2. UniProt-Reviewed Piscidins

Piscidins are also called pleurocidins, in reference to one of the first AMP sequences isolated from the mucosal cells of flounder [41]. To date, piscidins have been characterized in a variety of Teleost species, including cod (*Gadus morhua*), red bream (*Chrysophrys major*), sea bass (*Dicentrarchus labrax*), grouper (*Epinephelus coioides*), rainbow trout (*Oncorhynchus mykiss*), and striped bass (*Morone*), to cite a few. UniProt is a database that contains extensive descriptions of proteins and their role in various biological processes, molecular interactions, and pathways, as well as links to other useful databases [43]. According to UniProt, the pleurocidin protein family has about 360 entries (accessed on 14 March 2023). However, only 11 of them (Table 1) were reviewed by UniProt curators (Swiss-Prot). Swiss-Prot, founded in 1986, is included in the reviewed area of the UniProt Knowledgebase. Swiss-Prot is a high-quality, manually annotated, non-redundant protein sequence database that brings together experimental results, calculated features, and scientific conclusions. The TrEMBL part of the UniProtKB database was first made available in 1996 in response to the growing influx of data that was a direct result of genomic studies. The mature peptides and pro-domains of piscidin peptides from different fish species show little similarity [44]. The sequences of most piscidins’ mature active peptides are often predicted via homology or alignment with already known mature peptides from other fishes [45]. This is an important limitation to the study of these peptide families. Therefore, we decided to only study the 11 pleurocidin entries reviewed by UniProtKB curators.

In the InterPro database (accessed on 14 March 2023), the pleurocidin protein family comprised 17 structures determined via NMR and 333 Alphafold models. InterPro is a database that helps scientists analyze protein sequences by grouping them into families and making educated guesses about the presence of domains and key sites. The InterPro website (http://www.ebi.ac.uk/interpro, accessed on 14 March 2023) allows you to search for protein families, domains, and key sites; search for sequences; and browse InterPro annotations [46]. As a consortium, the databases that make up InterPro use predictive models (called signatures) contributed by other databases to properly categorize proteins [47]. The advantage of InterPro is that it combines the protein fingerprints of its member databases into a single searchable resource, leveraging the best features of each database to create a comprehensive diagnostic and research tool [48,49]. The pleurocidin IPR012515 (Pfam08108) motif was present on 360 proteins and 2 domain architectures. The first one comprised 358 proteins represented by Pleurocidin-like peptide WF3 of 61 amino acids (Q90VW7) from the winter flounder *Pseudopleuronectes americanus* [41], and the second one was with two proteins (A0A4Z2HBPO) represented by Dicentracin of 171 amino acids from *Liparis tanakae* (Tanaka’s snailfish) [50]. Detailed functional annotations and the addition of relevant gene ontology (GO) terms enhance the value of InterPro entries and enable the automatic annotation of millions of GO terms across all protein sequence databases. CATH cDD, HAMAP, MobiDB Lite, Panther, Pfam, PIRSF, PRINTS, Prosite, SFLD, SMART, SUPERFAMILY, and TIGRfams are just a few of the 13 member databases contributing signatures to InterPro [46,51]. The taxonomy entries of the sequences classified as pleurocidin families (IPR012515) by the InterPro database (accessed on 14 March 2023) can be visualized as an interactive sunburst view, where the weight of the segments is proportional to the number of sequences (https://www.ebi.ac.uk/interpro/entry/InterPro/IPR012515/taxonomy/uniprot/?cursor=source%3Ai%3A8267#sunburst, accessed on 14 March 2023). A total of 362 pleurocidin sequences from 94 fish species are annotated in this database (Supplementary Material Table S1). *Oreochromis niloticus* (Nile tilapia) is the Teleost species with the highest number of annotated sequences (18).

## 3. Evolutionary Diversity of Piscidins

**Figure 1:**
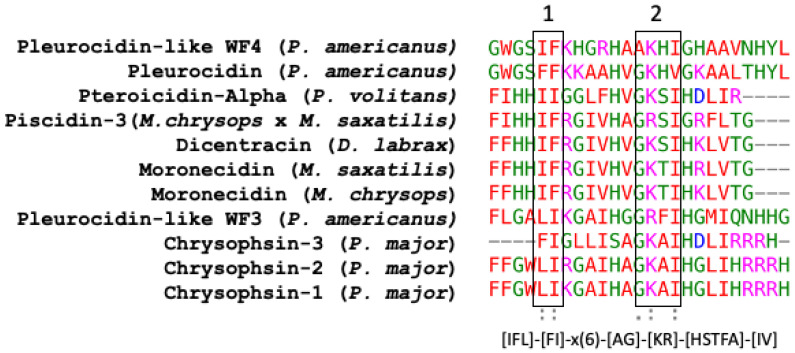
Alignment of the amino acid sequences of the mature active peptides from UniProt-reviewed piscidins (pleurocidin) using Clustal Omega (https://www.ebi.ac.uk/Tools/msa/clustalo, accessed on 14 March 2023). A colon (:) indicates strong homology, and a period (.) indicates weak homology, respectively, based on the Gonnet Pam250 matrix.

Piscidin is a class of peptides that is one of the most abundant AMPs in fish. In the course of studying AMPs unique to fish, we now know that certain peptides, originally named pleurocidins, and which we call piscidins, are present in a number of Teleost species from several families [21]. Piscidins are an extremely diverse family of AMPs, each with its own unique amino acid sequence. It was found that the piscidin peptides studied in detail varied in length and amino acid sequence depending on the fish species studied [44]. Piscidins are subject to both positive Darwinian selection and gene duplication, which would explain the wide range of peptides and low degree of sequence similarity among members of the piscidin family [52]. This suggests that various ecological and evolutionary influences have affected the evolution of piscidin peptides in different fish species [52]. Although there is some homology among piscidin peptides, variations in sequence and structure suggest that different piscidin peptides have evolved to perform specialized functions in different fish species [8]. Using the Hidden Markov Model and Seeded Guide Tree methods, the multiple sequence alignment tool Clustal Omega (accessed on 14 March 2023) created alignments between three or more sequences [53]. Asterisks denote identical residue, a colon indicates strong homology, and a period indicates weak homology, respectively, based on the Gonnet Pam250 matrix. The alignment of the 11 piscidin peptides shows that certain residues are conserved in two locations (squares 1 and 2 of Figure 1).

**Figure 2:**
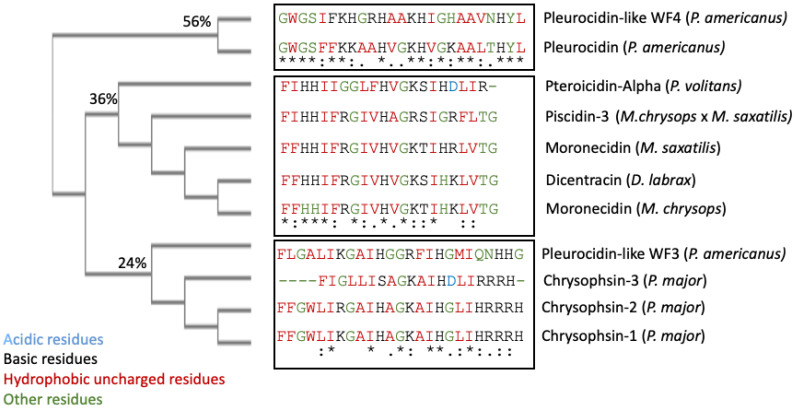
Clustal Omega guide tree obtained via sequence similarity of UniProt-reviewed mature piscidins. The percentages of identity among the clusters are shown in each branch. Acidic residues are colored in blue, basic residues in black, hydrophobic uncharged residues in red, and other residues in green, respectively. Asterisks (*) denote identical residues, a colon (:) indicates strong homology, and a period (.) indicates weak homology, respectively.

These two segments could be functionally important as they are conserved in the UniProt-reviewed piscidins. These regions have the signature [IFL]-[FI]-X-X-X-X-X-X-[AG]-[KR]-[HSTFA]-[IV]. However, no structural signatures have been identified in the mature peptides of piscidin. Guide trees are used to define the order in which pair-wise alignments are performed. The guide tree of the mature piscidin peptides from the UniProt-reviewed pleurocidins revealed three clusters of the active peptides. The first one comprised two pleurocidins from *P. americanus*: the Pleurocidin-like WF4 and Pleurocidin (Figure 2) with an identity of 56%. The second cluster comprised five pleurocidins: Pteroicidin-Alpha (*P. volitans*), Piscidin-3 (*M. chrysops* × *M. saxatilis*), Moronecidin (*M. saxatilis*), Dicentracin (*D. labrax*), and Moronecidin (*M. chrysops*), showing an identity of 36%. The third cluster comprised four peptides, the Pleurocidin-like WF3 (*P. americanus*) and Chrysophsin-1, 2, and 3 (from *P. major*), showing an identity of 24% (Figure 2).

It remains to be elucidated whether this grouping of mature piscidin peptides, based on their sequence similarity, could imply that the grouped peptides perform similar functions or act through similar mechanisms of action.

## 4. Piscidin Gene Arrangement, Processing, and Expression

Basal expression patterns of piscidin genes have been found to differ both within and between fish species. Furthermore, the expression levels of the different isoforms can vary widely within a species. Piscidin expression also begins early in fish development and continues to increase throughout the life cycle. For example, transcripts of pleurocidin-like genes have been found in winter flounder larvae as early as five days after hatching, and different pleurocidin-like genes are probably expressed at different developmental stages. Gill, skin, colon, brain, kidney, and spleen are just some of the tissues where these genes are consistently expressed [27]. In terms of cell types, piscidins are expressed by mast cells, rod cells, phagocytic granulocytes, and eosinophilic granulocytes. [54,55]. Piscidin gene expression has been shown to be altered following infection with a variety of pathogens [23]. Particularly, mucosal tissue contains piscidin peptides at levels that are lethal to pathogens [8]. 

**Figure 3:**
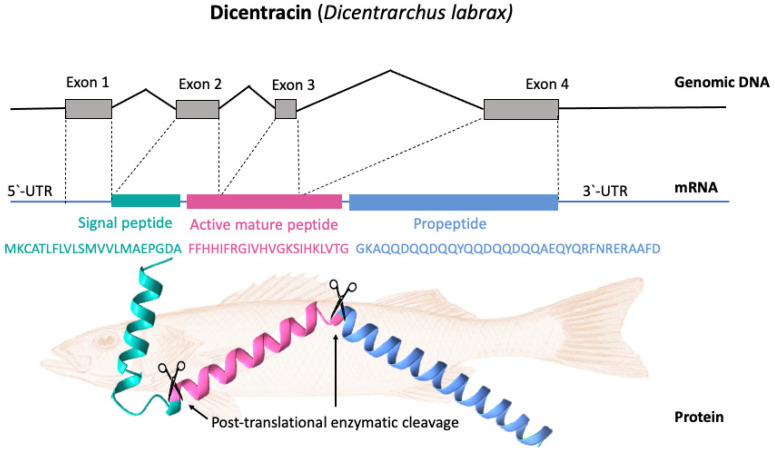
Gene organization and processing of Dicentracin piscidin from *Dicentrarchus labrax*. The precursor protein consists of a signal peptide, the active peptide, and the propeptide. The active mature Dicentracin peptide is obtained via post-translational cleavage of the pre-peptide.

Most piscidin genes encode a precursor with a signal peptide with 22 residues, a mature (active) peptide with 22–25 residues, and a variable C-terminal region [52,56]. Piscidins are encoded by four exons and three introns. The exons encode the signal peptide, full-length peptide, and propeptide. The 5′ untranslated region contributes to the formation of the first exon, which continues to the first nucleotide of the second exon. Exon 2 encodes the signal peptides, while exons 2, 3, and 4 encode the mature peptides. Exon 4 encodes the pro-domains, followed by the 3′ UTR. Exons in piscidin genes vary in size, with exon 4 being the largest [44]. Figure 3 shows, as an example, the gene organization of the Dicentracin peptide with four exons and three introns.

In general, the signal sequences and pro-regions typically found in AMPs are much better conserved than the mature active peptides themselves [57]. However, because AMPs are located at the interface between the host and a dynamic microbial biota, they are subject to significant positive selection for variation in many species [57]. This further reduces the already low degree of homology that exists between orthologous AMPs of even closely related species, and the fact that AMP sequences are often relatively short exacerbates the problem [58].

Several processes are involved in the production of active piscidin from the inactive precursor peptide. In the nucleus, the gene encoding piscidin is translated into messenger RNA (mRNA). In the second step (translation), the mRNA is taken to ribosomes in the cytoplasm, where it is converted into a peptide precursor [3]. To eliminate the signal sequence and produce an intermediate form, a signal peptidase cleaves the piscidin precursor protein [59]. Piscidins, once synthesized, are converted by enzymes from inactive precursor proteins to active peptides. Fish species may differ greatly in their processing procedures, although in most cases, the proteolytic cleavage of the precursor protein is involved [18]. In most cases, the mature piscidin peptide is released from the C-terminus of this intermediate by processing by local proteases, such as furin [60]. Cleavage occurs in the endoplasmic reticulum after the precursor peptide (which contains a signal sequence) is transported there and cleaved by signal peptidase to generate the propeptide. 

In the final step, the propeptide piscidin protein is transported into the skin mucus in the form of secretory granules. When piscidin protein reaches the dermal mucus, proteases can cleave the propeptide region, releasing the active peptide and allowing it to exert its effects [60]. The exact details of the processing of piscidin may differ somewhat depending on the species of fish in which it is found [59]. 

## 5. Structure

The ability of AMPs to produce membrane permeability is highly dependent on the secondary structure, which is highly susceptible to environmental factors [61]. This diversity is critical for effective defense against a variety of microbial pathogens [62]. The large number of anionic phospholipids in microbial membranes contrasts with the mostly zwitterionic elements of mammalian cell membranes, suggesting that electrostatic interactions play an important role in the interaction of AMPs with microbial membranes [63]. Moreover, AMPs are able to interact with hydrophobic parts of microbial architecture due to their tailored hydrophobicity. For this reason, the cationicity, amphipathicity, and secondary structure of AMPs may explain much of their function [63]. The positive, negative, neutral, or polar nature of the amino acids gives the peptide its overall charge. In the piscidin peptides reviewed by UniProt, the amino acids are predominantly hydrophobic rather than hydrophilic (Table 2), with a variable number (between six and nine) of cationic amino acids and the absence of negatively charged amino acids. The exception is Chrysophsin-3, which has one negatively charged amino acid (Asp). The rest of the amino acids in the piscidin peptide composition are neutral (Table 2). Peptides have a net positive charge at pH values below their isoelectric point (pI) and a net negative charge at pH values above their pI. The pI values of the piscidins ranged from 8.78 for Pteroicidin-alpha to 12.48 for Chrysophsin-2 (Table 2). The unique pI value of the target peptide can be used to study the purification process and pH conditions that can be used to separate the peptides from a mixture of peptides and/or proteins.

Mature peptides ranging from 18 to 46 residues in length are released from the signal peptide and pro-domain located at the N- and C-termini of piscidin proteins. Piscidin peptides have a much simpler tertiary structure than the best-studied AMPs; they lack cysteine residues, so they do not form disulfide bonds. The piscidin peptides share a number of structural features such as an alpha-helical structure and low molecular weight. These AMPs appear to be homologous to AMPs such as the cecropins found in the cecropia moth, *Hyalophora cecropia* [54]. A typical structural motif in antimicrobial peptides is the alpha helix, which is defined by a repeating pattern of hydrogen bonding between the carbonyl oxygen of one amino acid residue and the amide hydrogen of another, resulting in a compact, rod-like shape. Piscidins contain a cationic charge at physiological pH, which allows them to interact with negatively charged bacterial membranes [64]. 

An appropriate balance between hydrophobic and electrostatic contacts is crucial for the activity and mechanism of action of piscidins. The alpha-helical structure of piscidins is formed by a hydrophobic N-terminal region, while the positively charged C-terminal portion consists of cationic amino acid residues [23]. Due to its amphipathic nature, the alpha-helix can interact with both the hydrophobic lipid bilayer of microbial cell membranes and the negatively charged surface of the membrane [65]. 

**Figure 4:**
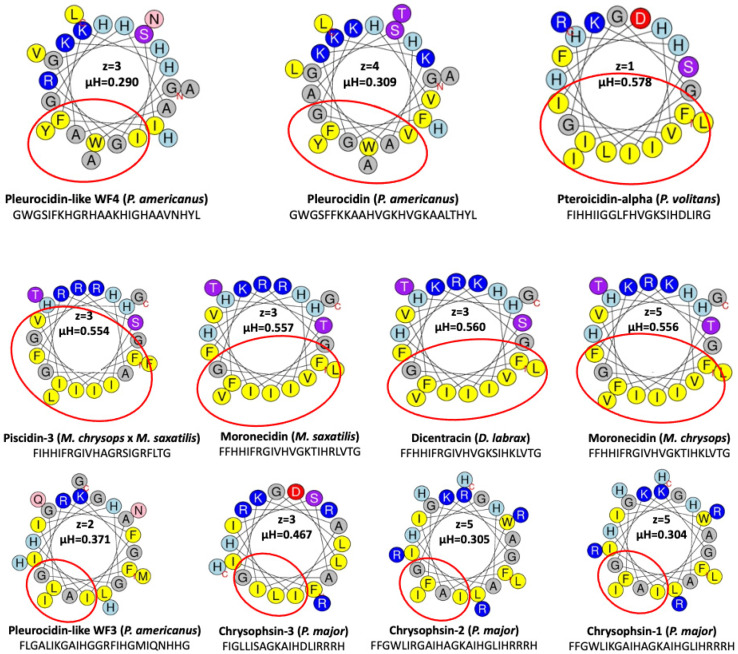
Helical wheel projections of UniProt-reviewed piscidin mature peptides. Properties such as hydrophobic moment (µH), net charge (z), and hydrophobic face (circled in red) were obtained from HeliQuest (https://heliquest.ipmc.cnrs.fr, accessed on 14 March 2023). The yellow residues are hydrophobic, while the blue residues are cationic. Polar acidic residues (D and E) are colored red, amine-group-containing residues (N and Q) are colored pink, hydroxi-amino acids (S and T) are colored purple, and glycine-containing residues are colored gray.

The helical wheel projections allow the visualization of the distribution of hydrophobic and polar residues with respect to the helical axis. The HeliQuest analysis tool (accessed on 14 March 2023) was used to obtain the Edmundson wheel projection of UniProt-reviewed piscidins. HeliQuest (https://heliquest.ipmc.cnrs.fr, accessed on 14 March 2023) determines properties such as the hydrophobicity, hydrophobic moment, net charge (z), and amino acid composition of the alpha-helices [66]. The alpha-helical properties of the piscidin peptides revealed that they have a net cationic charge at physiological pH and a hydrophobic face (Figure 4). 

The presence of basic amino acids such as arginine, lysine, and histidine is responsible for the net positive charge, while equal amounts of hydrophobic and hydrophilic amino acids contribute to the amphipathic character. According to this analysis, three piscidins (Moronecidin, Chrysophsin-2, and Chrysophsin-1) showed a net charge of +5, whereas Pleurocidin-like WF3 and Pteroicidin-alpha were the piscidin peptides with the lowest net charge of +2 and +1, respectively (Figure 4). All piscidin helices also had a hydrophobic side consisting of residues (Ala, Leu, Ile, Val, Met, Pro, Phe, Trp, and Tyr) adjacent on a helical wheel. According to this analysis, the piscidin Moronecidin (from *M. chryops*) had the greater value for the net charge combined with a large hydrophobic face of 10 hydrophobic residues and a hydrophobic moment µH of 0.556, whereas the alpha-helix of Pleurocidin-like WF4 (from *P. americanus*) showed the lowest value of the hydrophobic moment (µH) (Figure 4). According to the hydrophobic moment (µH) and the hydrophobic face of the helices, the value of µH varies from 0 to 3.26. When the value of µH is high, it indicates that the helix has an amphipathic structure perpendicular to its axis. All piscidins studied showed an amphipathic alpha helix with a hydrophobic moment µH between 0.3 and 0.5, supporting their potential antimicrobial activity.

## 6. Mechanisms of Action

The extracellular monolayer of eukaryotic cells typically contains uncharged zwitterionic amphiphiles (such as phosphatidylcholine lipids), whereas bacterial membranes are composed of 25% acidic lipids (i.e., phosphatidylserine and phosphatidylglycerol lipids and cardiolipin) [67]. The antibacterial capacity and cytotoxic activity of AMPs are influenced by their physicochemical properties, such as amphipathicity, hydrophobicity, and the number and position of cationic residues in the peptide sequences [68]. Potency and selectivity, on the other hand, are the result of a complex interplay of interrelated processes related to these properties and are therefore difficult to predict [69].

**Figure 5:**
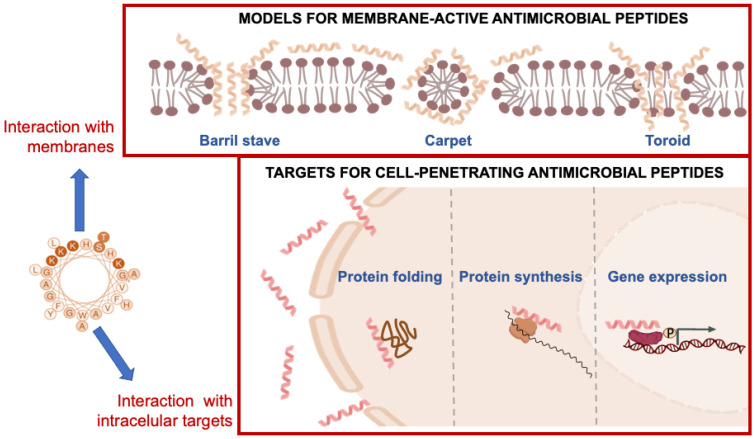
Mechanisms of action and model of membrane interactions proposed for piscidin peptides.

The interaction between piscidins and membranes is thought to be primarily electrostatic in nature, with the positively charged amino acid residues in the piscidin peptide interacting with the negatively charged phosphate head groups of the lipids in the membrane [63]. This interaction can lead to the formation of transient pores or channels in the membrane, which disrupt the membrane barrier function and allow ions and other cellular components to leak out (Figure 5). 

Amphiphilic peptides are thought to bind initially to a membrane in a flat configuration. If the concentration is high enough, the membrane can be damaged in a non-specific manner, as in the carpet mechanism. On the other hand, it has been shown that oligomeric holes can form when peptide molecules rearrange after being inserted into a membrane. It is important to distinguish between the “toroidal wormhole” model, in which anionic lipids contribute to the lining of the pore, and the “barrel-stave” model, in which the pore consists entirely of peptide molecules (Figure 5). Although the exact mechanism of action of peptides is not yet clear, all piscidin peptides studied might have membrane-binding and disrupting properties, leading to the formation of transient pores or channels in the membrane and ultimately microbial death [63]. The hydrophobic component of piscidin peptides can also result in partial or complete penetration into the lipid bilayer, resulting in the disruption, destruction, or fragmentation of the microbial membrane [38]. Several data suggest the possibility that these AMPs may not only create pores but also interfere with cellular processes such as protein and nucleic acid synthesis and enzymatic reactions. The linear alpha-helical structure and a number of positive charges raises the possibility that piscidins can act against bacteria by forming pores that permeabilize the bacterial membrane [70] (Figure 5).

Although Moronecidin and Piscidin-3 have a stronger affinity for LPS, they are deposited in both phospholipid and LPS monolayers. The preferential deposition of the peptides in the outer LPS layer of the bacterial membrane is thought to be responsible for their selectivity against Gram-negative bacteria [71]. The mature 22-amino acid peptide Dicentracin exerts its effects by forming pores in the membranes of bacteria [23]. The mode of action of Pleurocidin is thought to involve the formation of transmembrane channels in the outer bacterial membrane, resulting in the permeability of the phospholipid bilayer [72]. The bacteriostatic and bactericidal effects of Pleurocidin are quite broad (Table 3) but have been shown to be somewhat attenuated against *Leucothrix mucor*, *Pseudomonas aeruginosa*, and *Serratia marcescens* [73]. The orientation and conformation of peptides in lipidic environments are also affected by the peptide to lipid molar ratio [38]. When the peptide-lipid ratio is low, the peptide binds perpendicular to the membrane. However, when the amount of peptide is increased, peptides convert to a tilted form that changes their orientation with respect to the lipid surface and crosses the membrane either partially or completely. As a result, other models such as the barrel-stave model, the carpet model, and the toroidal pore model have been developed to explain membrane permeabilization [72]. Both Moronecidin and Piscidin-3 have been reported to adopt an helical structure in SDS and DPC micelles, but in the presence of lipid bilayers, they adopt a kinked structure with a central glycine [74]. It cannot be ruled out that head-tail dimers or oligomers form in the presence of bacterial membranes. However, it has been suggested that increasing the net positive charge of the low-hydrophobicity peptide above a threshold level may result in high antibacterial activity with low cytotoxicity [38]. 

**Figure 6:**
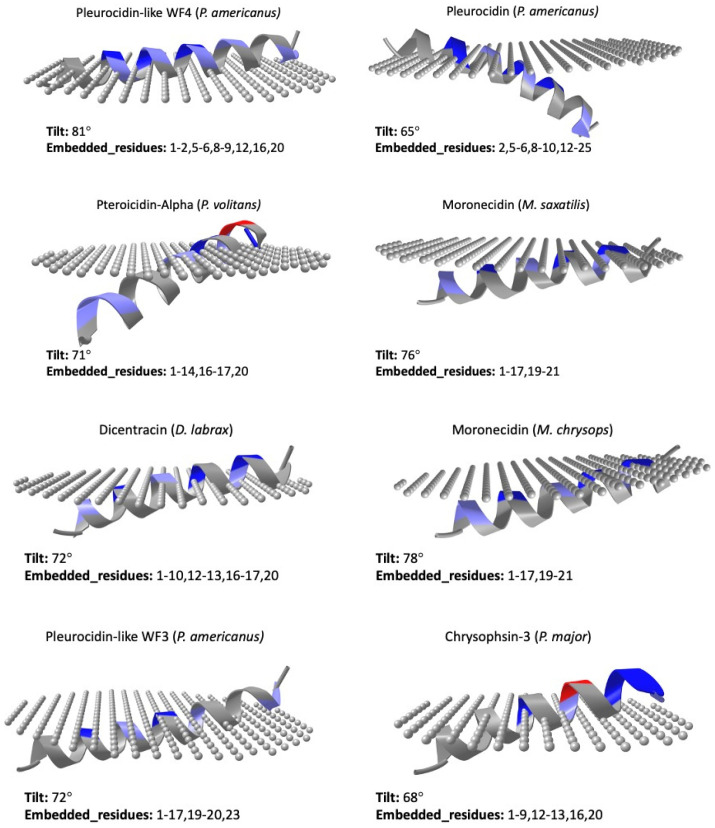

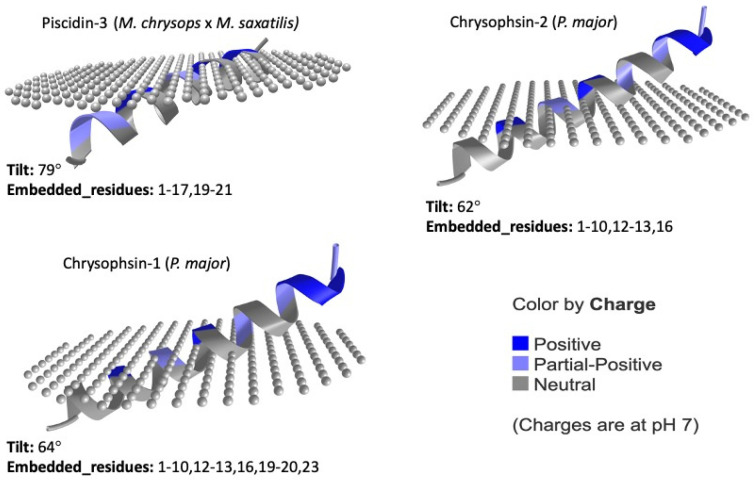
Predicted orientation of the UniProt-reviewed mature piscidins using the prediction tool PPM 2.0 of the Orientations of Proteins in Membranes (OPM) database (https://opm.phar.umich.edu, accessed on 14 March 2023). The tilt angle and number of embedded residues of peptide structures in membranes is provided by the PPM 2.0 server.

To investigate the possible mechanism of action of the piscidins deposited in the UniProt database under the reviewed category, we used the prediction tool of the Orientations of Proteins in Membranes (OPM) database (https://opm.phar.umich.edu, accessed on 14 March 2023). The PPM 2.0 server (accessed on 14 March 2023) provides rotational and translational positions in membranes of transmembrane and the visualization of calculated proteins structures in membranes [87]. The predicted orientations of the UniProt-reviewed mature piscidins are shown in Figure 6.

Using this prediction tool, active piscidin peptides, which are positively charged, interact with lipids of the outer cytoplasmic membrane, which are negatively charged, with tilt angles between 60° and 80°. The tilt closest to 90° was predicted for the Pleurocidin-like WF4 from *P. americanus* (tilt: 81). In contrast, the active mature peptide of Chrysophin-2 (from *P. major*) was the most vertically inclined (tilt 62°) peptide. Three piscidins (Moronecidin, Chrysophsin-2, and Chrysophsin-1) showed a net charge of +5, whereas Pleurocidin-like WF3 and Pteroicidin-alpha were the piscidin peptides with the lowest net charges of +2 and +1, respectively (Figure 4). Hemolytic activity is often related to helix formation, whereas antibacterial activity is associated with structural flexibility. In general, the closer the tilt angle is to 90°, the less active the peptide is against erythrocytes [88]. According to the membrane penetration of the piscidin peptides, the Moronecidin (from *M. chrysops*), Pleurocidin-like WF3 (*P. americanus*), and Piscidin-3 (from *M. chrysops* × *M. saxatilis*) showed almost all residues embedded in the simulated membrane (Figure 6). This is consistent with the larger hydrophobic face of these peptides (Figure 4). 

The presence and position of negatively charged residues in the alpha-helix may prevent favorable interaction with the membrane in regions near these residues. However, they could also play an important role in the orientation of the alpha-helix by favoring interaction with hydrophobic residues on the opposite side of this charge. This could allow greater penetration of these residues into positions longitudinally farther from the negatively charged residue. This could be the case for peptides such as Pteroicidin-alpha (*P. volitans*) and Chrysophin-3 (*P. major*), which have a higher number of residues embedded in the membrane in a position opposite to the negatively charged residue in the longitudinal direction (Figure 6).

On the other hand, when positively charged residues are well grouped on one side of the alpha helix, a more favorable interaction with the negatively charged membrane occurs, especially when these residues are arranged longitudinally along the alpha-helix. The tilt angle of the peptide with respect to the membrane is then closer to 90 degrees. However, if the positively charged residues are further apart in the Edmundson helix projection, coinciding with a lower hydrophobic moment of the alpha-helix, a flat orientation of the peptide may not be favored, and the orientation tilt angles may be less than 90 degrees. This is exactly what happens in the case of Pleurocidin-like WF4 (*P. americanus*), where the positively charged residues are much better grouped in the Edmundson helix projection (Figure 4), resulting in a tilt value of 81 degrees (Figure 6). In contrast, for the Chrysophsin-2 (*P. major*) peptide, these positively charged residues are dispersed and separated in the same projection (Figure 4), resulting in a tilt inclination value of 62 degrees (Figure 6). Piscidins 1 and 3 are able to translocate bacterial cell membranes and adhere to target sites to induce cell death due to their binding and disruption properties. The increased structural flexibility and lack of amphipathicity could also be due to the high histidine concentration in the piscidin peptide Of-Pis1 [38]. Histidines are also thought to confer on AMPs the potential to reorganize membranes by altering lipid distribution in response to heterogeneous membrane composition as well as changing their orientation along the lipid bilayer and their conformation and insertion depth [38,89].

Marine AMPs are able to tolerate high salinity due to their evolutionary adaptations. Piscidins are unique in that their antibacterial activity is not affected by high salt levels. It has been shown that the antibacterial activity of piscidin peptides, as well as their resistance to changes in pH and salinity, is significantly affected by the position and amount of histidines in the peptide [38]. Amino acid residues such as histidine, aspartic acid, and glutamic acid are protonated under acidic conditions, and this structure–function relationship is largely responsible for the pH-dependent action of AMPs. Thus, the fate of the AMP molecule in its interaction with the predominantly anionic microbial membrane is determined by whether or not these residues are protonated, thereby enhancing the cationic properties of the molecule as a whole [90]. The structural–functional relationship of AMPs also affects their potential to disrupt microbial membranes by binding to metal ions such as Zn^2+^, which then form peptide–membrane or peptide–peptide salt bridges [91,92]. The electrostatic connections between peptides and the membrane can be disturbed at high salt concentrations, since cations can bind to the negative charges of the membrane. Metal-binding sites of peptides are usually surrounded by hydrophilic residues that can be structurally disassembled [93]. An amino-terminal copper and nickel motif (ATCUN) is found in some piscidins and is responsible for Cu^2+^ coordination [94]. For bacteria and fungi to survive and become virulent, they must effectively take up Zn^2+^ and Cu^2+^ [95]. Two of the piscidin peptides reviewed by UniProt (Mononecidin and Piscidin-3) contain ATCUN motifs [96]. Both peptides coordinate Cu^2+^ with picomolar affinity when exposed to aerobic conditions by using their amino-terminal copper- and nickel-binding (ATCUN) motifs. In an aerobic environment, these copper-ATCUN complexes can act as sources of oxidative stress and increase the lethality of the peptides against bacteria [78]. The ability of these peptides to generate radicals that nick DNA in the presence of Cu^2+^ is related to their bactericidal activity [92]. Reactive oxygen species (ROSs) are generated by Fenton-like chemical reactions during metal binding. Therefore, the ATCUN-AMP sequence is thought to represent complicated but efficient antibacterial machinery in vertebrates by producing ROSs [93,97]. To gain a better understanding of the piscidins’ mode of action and to better assess their potential selectivity, we used the bioinformatics tool *Me*Bi*Pred*, at https://services.bromberglab.org/mebipred/home (accessed on 15 March 2023). This approach, based on sequence information alone, has greater than 80% accuracy in identifying proteins that bind ligands containing metal ions. It can be used for the analysis of newly identified proteins or peptides with no known homologs [98]. Metal-binding peptides identified using the machine-learning-based approach showed that two piscidin peptides (Pleurocidin-like WF4, from *P. americanus*, and Chrysophsin-3, from *P. major*) were able to bind zinc metal, whereas the piscidin peptides Chrysophsin-2 and Chrysophsin-1 (from *P. major*) were predicted to bind copper metal (Table 4).

The anticancer activity of some piscidins is enhanced upon metal binding. Metallized peptides destroy lipid membranes both mechanically and chemically [90]. Piscidin-3 is particularly efficient at introducing its metallated motif into bilayers, leading to the formation of water clefts in the hydrocarbon region and positioning Cu^2+^ in close proximity to the double bonds of the acyl chains, which is necessary for the oxidation of these bonds [90]. This encourages the development of more effective peptide-based cancer therapeutics, with the goal of metallating AMPs to increase their mechanistic range. 

## 7. Function

Piscidins have shown potent antimicrobial activity in vitro against a variety of pathogens, including Gram-positive and Gram-negative bacteria, multidrug-resistant bacteria, viruses, fungi, cancer cells, and even certain parasites [23]. In fish, piscidins are stored in the granules of phagocytic granulocytes and transported to pathogen-containing phagosomes during phagocytosis, suggesting that fish may utilize piscidins as antimicrobial agents in vivo to kill invasive infections. After the active piscidin peptide is synthesized, it can interact with the membranes of microorganisms, compromising their function and eventually killing the cells [38]. The piscidin peptides reviewed by UniProt showed distinct antimicrobial activity against common bacterial strains and fungi, such as *E. coli*, *S. aureus*, *P. aeruginosa*, and *C. albicans*. In particular, they also exhibited antimicrobial activity against fish and oral pathogens, such as *A. salmonicida* and *S. mutans*, respectively (Table 3).

Certain piscidins have been shown to bind to enzymes involved in peptidoglycan production, inhibiting bacterial cell wall synthesis and leading to bacterial cell death [99]. The ability of piscidins to interact with microbial membranes is also important for their broad-spectrum antimicrobial activity, as microbial membranes are a conserved feature across a wide range of microbial species [100]. Hydrophobic parts of the peptide can penetrate the membrane and create pores or break lipid bilayers, while the cationic charge of the peptide allows it to adhere to negatively charged microbial membranes [101,102]. Ultimately, this causes the microbial cell to lyse and die. It is important to understand why AMPs act selectively on bacteria and not on host cells. The answer to this question will shed light on the mechanism of action of AMPs and pave the way for the development of more targeted peptide antibiotics [95]. Multiscale molecular dynamics simulations can help clarify how membrane composition affects the behavior of a transmembrane pore formed by peptides [70]. 

The database of antimicrobial activity and structure of peptides (DBAASP) web server (https://dbaasp.org/home, accessed on 14 March 2023) can be used for the prediction of the antibacterial activity of peptides. DBAASP (accessed on 14 March 2023) provides several tools, including a rigorous multifactor analysis of key physicochemical properties, to support the creation and optimization of de novo peptides with the appropriate biological activity [103]. Because of these capabilities, DBAASP has become a popular tool for building AMP predictive models that can tell you whether or not an individual peptide is active against a particular microbial strain.

**Figure 7:**
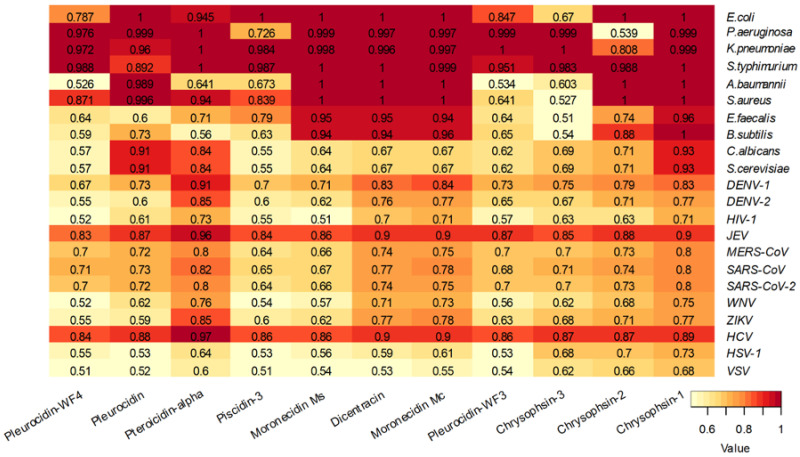
Heatmap of prediction of antibacterial, antifungal, and antiviral activity of piscidin peptides reviewed by UniProt using the DBAASP web server at https://dbaasp.org/home (accessed on 14 March 2023). Values indicate antimicrobial score (from 0 to 1).

The analysis of the piscidin peptides reviewed by UniProt showed that these peptides are predicted to be effective against bacteria rather than fungi or viruses, with the exception of Japanese encephalitis virus and hepatitis C virus (Figure 7). The piscidin peptides Pleurocidin WF3 and Chrysophsin- 3 are predicted to be less active against bacteria (Figure 7).

**Figure 8:**
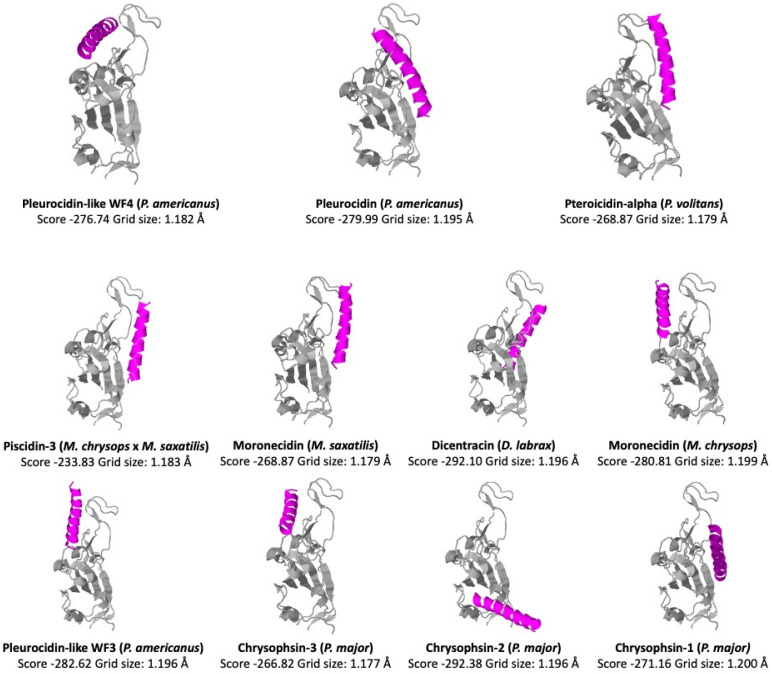
Docking analysis of UniProt-reviewed piscidin peptides and the region-binding domain (RBD) of the SARS-CoV-2 Spike protein using the COVID19 docking server (https://ncov.schanglab.org.cn, accessed on 14 March 2023). Scores and grid sizes were calculated using the CoDockPP (CoDockPP Server) docking engine.

AMPs are promising reagents against SARS-CoV-2. Several AMPs isolated from fish have been shown to have antiviral activity, but the underlying cellular mechanisms remain poorly understood and require further research [104]. Piscidins isolated from hybrid striped bass were found to be effective against channel catfish virus [105]. In addition, zebrafish beta-defensin 2 (zfBD2) significantly reduced the carp spring viremia of carps [106]. In addition, Hepcidin-2 from spotted scat (*Scatophagus argus*) was active against Siniperca chuatsi rhabdovirus (SCRV) and largemouth bass Micropterus salmoides reovirus (MsReV) in epithelioma papulosum cyprini (EPC) and grass carp fin (GCF) cells [107]. To determine whether the UniProt-reviewed piscidins could be active against SARS-CoV-2 virus, we performed a docking analysis of the region binding domain (RBD) of the SARS-CoV-2 Spike protein (Figure 8). We used the COVID19 docking server [108] (https://ncov.schanglab.org.cn), accessed on 15 March 2023, for the prediction of the binding and orientation of the peptides. The scores and grid sizes were calculated using the CoDockPP (CoDockPP Server) docking engine. 

By interacting with the hydrophobic surfaces of the virus, peptides with positive interfacial hydrophobicity can prevent the fusion and entry of the virus. According to this analysis, the higher docking scores of −292.10 and −292.38 were obtained for Dicentracin (from *D. labrax*) and Chrysophsin-2 (from *P. major*), respectively. The lowest score (−233.83) was obtained for the Piscidin-3 peptide (from *M. chrysops* × *M. saxatilis*). The piscidin scores with higher negative values indicated a potential ability to bind to the RBD of the Spike protein. We compared these values with the score of −308.29, obtained for the human cathelicidin peptide LL-37 with same docking analysis [109]. Previous studies have reported the docking analysis of piscidin peptides Pleurocidin (from *P. americanus*) and Chrysophsin-2 (from *P. major*) with the RDB domain of MERS, where Pleurocidin obtained the highest score based on cluster size [110].

Recently, it has become clear that the action of some peptides in fighting infections is due to their broad immunomodulatory activities rather than their limited direct antibacterial activities in mammals. The interaction of some piscidin peptides with membranes can also have immunomodulatory effects, including the activation of immune cells and the regulation of cytokine and chemokine production [111,112]. This makes piscidins attractive candidates for the development of novel antimicrobial and immunomodulatory therapeutics [20]. Specifically, piscidins have been shown to stimulate the proliferation and activation of T cells, including both CD4+ and CD8+ T cells. Piscidins have also been shown to stimulate the production of cytokines and chemokines via immune cells, including interleukin-1 (IL-1), interleukin-6 (IL-6), tumor necrosis factor-alpha (TNF-alpha), and interferon-gamma (IFN-gamma) [30,112].

Immunomodulatory peptides are a broad category of bioactive peptides that includes many different types of molecules, all of which act through different modes of action [113,114]. Some antimicrobial peptides have recently become known for their immunomodulatory effects [115]. The modulation of the innate immune response is an effective technique to advance peptides as novel anti-infectives, as demonstrated by the protective effects of briefly synthesized peptides against infections in vivo [116]. Especially in the case of pandemics, it would be ideal for vaccines to contain immunomodulatory peptides as adjuvants [113,117]. Immunomodulatory peptides have the potential to be used both as useful additives for novel functional food preparations and as potential agents for drug development to treat a variety of diseases resulting from impaired immune system function [113]. Immunomodulatory peptides have a broad spectrum of activity that requires multiple tests to evaluate their efficacy. For example, Piscidin 1, from hybrid striped bass, was found to inhibit the production of pro-inflammatory cytokines such as IL-8 and TNF-alpha in lipopolysaccharide (LPS)-stimulated macrophages. Similarly, Piscidin 4, from the black rockfish, was found to inhibit the production of IL-1beta and IL-6 in LPS-stimulated macrophages [118].

One of the most difficult challenges in developing immunomodulatory peptides as drugs is determining how the peptides interact with and stimulate cells of the immune system [115,119]. Immunomodulatory peptides have been shown to target numerous receptors and activities within cells, depending on the cell type and amino acid sequence [115]. The recently developed tool, VaxinPAD (https://webs.iiitd.edu.in/raghava/vaxinpad, accessed on 14 March 2023) can be very helpful because it uses current knowledge about immunomodulatory peptides and a bioinformatics approach to predict how they will act in living organisms. This tool uses immunomodulatory (A-cell epitopes) and non-immunomodulatory peptides to train Support Vector Machine (SVM) models and is able to predict A-cell epitopes using a variety of sequence-based features [120]. Using VaxinPAD, we could predict whether the piscidin peptides are immunomodulatory (Table 5). We also used other tools, such as ProInflam [121], PreAIP [122], and AllerTOP [123], to study the pro-inflammatory (http://metagenomics.iiserb.ac.in/proinflam, accessed on 14 March 2023), anti-inflammatory (http://kurata14.bio.kyutech.ac.jp/PreAIP, accessed on 14 March 2023), or allergenic (https://www.ddg-pharmfac.net/AllerTOP, accessed on 14 March 2023) activity of the peptides, respectively (Table 5). 

In addition to their pro-inflammatory effects, piscidins have also shown anti-inflammatory activity. Chrysophsin-1, for example, has anti-inflammatory properties by inhibiting TNF-α secretion [124,125].

When tissues are damaged by pathogens, toxins, trauma, heat, or other causes, natural inflammatory responses occur. Chronic autoimmune and inflammatory diseases such as multiple sclerosis, cancer, rheumatoid arthritis, asthma, and psoriasis are affected by this response. The immunotherapeutic potential of anti-inflammatory peptides (AIPs) has a variety of clinical applications, including the induction of regulatory T cells and the suppression of antigen-specific Th1 responses [126]. Prior to in vitro testing, it would be helpful to classify potential anti-inflammatory peptides using novel in silico predictors. 

According to these bioinformatic tools, all piscidin peptides examined are anti-inflammatory (Table 5). The piscidin peptides Dicentracin, Moronecidin (from *Morone chrysops*), and Pleurocidin-like WF3 are of particular interest because they showed immunomodulatory properties, in combination with pro-and anti-inflammatory activities, without the potential to cause allergic reactions (Table 5). Chrysophin 2 was not allergenic but was only predicted to have anti-inflammatory properties (Table 5).

Therapeutic drugs that efficiently and selectively target tumor cells, such as anticancer peptides (ACPs), are very promising [127]. There are many known mechanisms of action of ACPs: they can damage the cell membranes of malignant cells and induce apoptosis by entering mitochondria and triggering the release of cytochrome c [128]. They can also affect signal transduction and cell cycle regulation by binding to specific membrane receptors [129,130]. The xDeep-AcPEP regression method (accessed on 14 March 2023) is a breakthrough approach to predicting the bioactivity of anticancer peptides using a convolutional neural network and multi-task learning [131]. Six different types of tumor cells (breast, colon, cervix, lung, skin, and prostate) were used to train a series of cancer-specific models using the CancerPPD datasets. The applicability domain (AD) of each model was established to estimate the uncertainty of a prediction for an unknown case. Three piscidin peptides (Pleurocidin, Chrysophsin-2, and Chrysophsin-1) were out of this applicability domain of the prediction tool (Table 6).

AMPs produced by marine animals are very effective against many different types of cancer cells and diseases [132]. Peptides such as those found in hybrid striped bass and their homologs in other fish are particularly promising. The antitumor efficacy of piscidins has been demonstrated in a variety of cancer cell lines, including A549, U937, HT1080, HeLa, HL60, MDA-MB-468, SKBR3, MCF7, T47-D, MDA-MB-231, MCF7-TX400 (paclitaxel-resistant MCF7), 4T1, and MCF7 [133,134,135].

In breast cancer, the piscidin peptide Pleurocidin-like WF3 had the lowest concentration (6.9 µM), in contrast to the higher concentrations of Pleurocidin-like WF4 (34.1 µM) and Piscidin-3 (33.2 µM) predicted against this type of cancer. However, this piscidin peptide required a much higher concentration to be active against colon (116.1 µM) and prostate (100.5 µM) cancers. For skin cancer, all piscidin peptides that were active showed similar levels between 4.5 µM and 8.2 µM. For prostate cancer, the peptide Piscidin-3 was predicted to be the least active peptide (226.7 µM), in contrast to Chrysophsin-3 (19.3 µM). However, Chrysophsin-3 was the least active in cervical cancer (requiring a concentration of 95.0 µM) compared to Pleurocidin-like WF4 (10.7 µM). This predictive tool illustrates the broad spectrum of activity of piscidin peptides against different cancers, as previously reported for piscidin peptides such as Pleurocidin from *Paralichthys dentatus*, or Piscidin 5-like from *Larimichthys crocea* [136]. Amino acid sequence and structure have significant implications for the efficacy profile of peptide drugs [132]. 

## 8. Conclusions and Future Trends

Peptides derived from piscidins have some characteristics that make them very interesting. A common feature of all of them is their alpha-helical structure, which is often amphipathic. The great stability attributed to these alpha-helices under high-salt conditions, and in the presence of metals, makes them very promising as antiviral agents. This makes sense because the ocean harbors a greater diversity of microorganisms, including viruses, which are more abundant than in terrestrial habitats.

The function of the C-terminal portion of piscidins is poorly understood. This portion probably acts to neutralize the antimicrobial or immunomodulatory activity of the mature active peptides during processing. Despite the number of piscidins deposited in the UniProt database, the great diversity in size and the lack of knowledge about the processing of their mature peptide in many of them have led us to focus our attention on the reviewed and manually curated piscidin peptides. Bioinformatics tools capable of predicting different aspects of peptide properties are continuously developing, and they are expected to predict the cleavage point of the mature peptide of piscidins with greater precision. Thanks to bioinformatics tools, we can study the physicochemical characteristics of their structure and their ability to bind metals; predict their biological activity, both antimicrobial and immunomodulatory; and conduct molecular docking studies to predict possible binding to viral receptors or other proteins of interest. However, to confirm the mechanism of action suggested by bioinformatics prediction techniques, specific in vitro assay studies are required. The limitations of these bioinformatic studies are mainly the lack of wet-lab validations for some of the antibacterial, anticancer, and immunomodulatory effects of piscidin peptides.

The potential of AMPs as alternatives to conventional antibiotics or antivirals in aquaculture, as immunological modulators, and even as oncological treatments or immunogenic drugs has attracted the interest of the scientific community. However, the functional aspects of host defense peptides in fish have not received nearly as much attention as their equivalents in non-fish. Piscidins can be used directly to defend against pathogens as an active ingredient in drugs or indirectly to modulate their immune systems and even as adjuvants to enhance the effects of vaccinations. In aquaculture, it is possible that antimicrobial drugs used to treat microbial diseases reduce fish mortality, which leads to increased fish production. To know the commercial value of these compounds and their use in food channels, it is important to understand the biological activity of peptides from fish.

Concerning the biotechnological production of piscidins, the chemical synthesis of the mature active peptides is expensive and therefore only suitable for preliminary screening methods, as large quantities of peptides need to be produced for physiological studies and clinical trials. An alternative technique for cost-effective production is in vitro expression using recombinant DNA technology in bacterial and/or yeast systems.

In biomedicine, advances are expected with functionalized peptides with lipids, metallic particles, or encapsulated for smart delivery to target tissues. Metal-based compounds represent promising potential new drugs for different diseases. The study of piscidin peptides could help design new therapeutics and materials, inspired by nature, for use in the areas of drug-resistant bacteria, neurological disorders, cancer, inflammation, and biomedical imaging. The amino-terminal copper- and nickel-binding (ATCUN) motif has garnered much interest due to the tuning effects created by different coordination geometries. Nanotechnology is expected to contribute to the functionalization of these peptides into metallic particles or vehicles for medicines that will help increase our arsenal of antibiotics.

## Figures and Tables

**Table 1:** Pleurocidin protein family found in InterPro database (accessed on 14 March 2023), reviewed by UniProt curators (Swiss-Prot), and length of their active peptides obtained from each entry.

Accession	Name	Species	Length
Q90ZX8	Pleurocidin-WF4	*Pseudopleuronectes americanus* (Winter flounder)	25
P81941	Pleurocidin	*Pseudopleuronectes americanus* (Winter flounder)	25
P0DUJ5	Pteroicidin-alpha	*Pterois volitans* (Red lionfish)	21
P0C006	Piscidin-3	*Morone chrysops* × *Morone saxatilis* (White bass × Striped bass)	22
Q8UUG0	Moronecidin Ms	*Morone saxatilis* (Striped bass)	22
P59906	Dicentracin	*Dicentrarchus labrax* (European seabass)	22
Q8UUG2	Moronecidin Mc	*Morone chrysops* (White bass)	22
Q90VW7	Pleurocidin-WF3	*Pseudopleuronectes americanus* (Winter flounder)	25
Q90VW7	Chrysophsin-3	*Pagrus major* (Red seabream)	20
P83546	Chrysophsin-3	*Pagrus major* (Red seabream)	25
P83545	Chrysophsin-1	*Pagrus major* (Red seabream)	25

**Table 2:** Number of amino acids that are hydrophobic, hydrophilic, positive, negative, and neutral in the piscidin peptide composition. Isoelectric point (pI) is the pH at which a molecule carries no net electric charge or is electrically neutral. Mw is the theoretical molecular weight of the peptide.

Piscidin Peptide	Hydrophobic	Hydrophilic	Positive	Negative	Neutral	pI	Mw
Pleurocidin-like WF4	15	10	8	0	17	10.29	2765.14
Pleurocidin	16	9	7	0	18	10.18	2711.17
Pteroicidin-Alpha	13	8	6	1	14	8.78	2409.87
Piscidin-3	14	8	6	0	16	12.30	2491.93
Moronecidin *M.s*	13	9	7	0	15	12.01	2572.06
Dicentracin	13	9	7	0	15	11.17	2530.02
Moronecidin *M.c*	13	9	7	0	15	11.17	2544.05
Pleurocidin-like WF3	16	9	6	0	19	11.00	2682.15
Chrysophsin-3	12	8	6	1	13	11.71	2286.75
Chrysophsin-2	16	9	9	0	16	12.48	2920.47
Chrysophsin-1	16	9	9	0	16	12.31	2892.46

**Table 3:** Antimicrobial activity of UniProt-reviewed piscidin peptides with reported MIC values < 20 µM.

Name	Antimicrobial Activity	References
Pleurocidin-WF4	*A. salmonicida*, *S. enterica*, *P. aeruginosa*, *E. coli*, *S. epidermidis*	[75]
Pleurocidin	*P. aeruginosa*, *E. coli*, *S. epidermidis*, *S. aureus*, *C. albicans*	[76]
Pteroicidin-alpha	*L. monocytogenes*, *E. faecalis*, *S. aureus*, *E. coli*, *A. salmonicida*, *V. vulnificus*	[77]
Piscidin-3	*C. difficile*, *M. furfur*, *T. beigelii*, *C. albicans*	[78,79]
Moronecidin Ms	*C. difficile*, *S. aureus* (*MRSA*), *M. furfur*, *T. beigelii*, *C. albicans*	[78,79,80]
Dicentracin	*E. coli*, *S. aureus*, *S. epidermidis*, *C. albicans*, *C. tropicalis*	[81]
Moronecidin Mc	*M. furfur*, *T. beigelii*, *C. albicans*	[82]
Pleurocidin-WF3	*E. coli*, *S. aureus*, *P. aeruginosa*	[83]
Chrysophsin-3	*S. mutans*, *E. faecalis*	[84]
Chrysophsin-2	*E. coli*, *S. aureus*	[85]
Chrysophsin-1	*S. mutans*, *S. sanguinis*, *S. sobrinus*, *L. acidophilus*, *E. faecalis*	[86]

**Table 4:** Potential ability of piscidins to bind metals, as predicted using the bioinformatics tool *Me*Bi*Pred*, at https://services.bromberglab.org/mebipred/home (accessed on 15 March 2023).

Piscidin Peptide	Ca^2+^	Co^2+^	Cu^2+^	Fe^2+^/Fe^3+^	K	Mg^2+^	Mn^2+^	Na	Ni^2+^	Zn^2+^
Pleurocidin-like WF4	-	-	-	-	-	-	-	-	-	**+**
Pleurocidin	-	-	-	-	-	-	-	-	-	-
Pteroicidin-Alpha	-	-	-	-	-	-	-	-	-	-
Piscidin-3	-	-	-	-	-	-	-	-	-	-
Moronecidin *M.s*	-	-	-	-	-	-	-	-	-	-
Dicentracin	-	-	-	-	-	-	-	-	-	-
Moronecidin *M.c*	-	-	-	-	-	-	-	-	-	-
Pleurocidin-like WF3	-	-	-	-	-	-	-	-	-	-
Chrysophsin-3	-	-	-	-	-	-	-	-	-	**+**
Chrysophsin-2	-	-	**+**	-	-	-	-	-	-	-
Chrysophsin-1	-	-	**+**	-	-	-	-	-	-	-

**Table 5:** Immunomodulatory, proinflammatory, anti-inflammatory, and allergenic properties of piscidin peptides from the UniProt-reviewed category using the bioinformatics tools VaxinPAD (https://webs.iiitd.edu.in/raghava/vaxinpad, accessed on 14 March 2023), ProInflam (http://metagenomics.iiserb.ac.in/proinflam, accessed on 14 March 2023), PreAIP (http://kurata14.bio.kyutech.ac.jp/PreAIP, accessed on 14 March 2023), and AllerTOP (https://www.ddg-pharmfac.net/AllerTOP, accessed on 14 March 2023), respectively.

Piscidin Peptide	Immunomodulatory	Pro-Inflammatory	Anti-Inflammatory	Allergenic
Pleurocidin-like WF4	No	Yes	Yes	No
Pleurocidin	No	Yes	Yes	Yes
Pteroicidin-Alpha	No	Yes	Yes	No
Piscidin-3	No	Yes	Yes	Yes
Moronecidin *M.s*	Yes	No	Yes	No
Dicentracin	Yes	Yes	Yes	No
Moronecidin *M.c*	Yes	Yes	Yes	No
Pleurocidin-like WF3	No	Yes	Yes	No
Chrysophsin-3	No	Yes	Yes	No
Chrysophsin-2	No	No	Yes	No
Chrysophsin-1	Yes	Yes	Yes	Yes

**Table 6:** Potential anticancer activity of UniProt-reviewed piscidins against 6 different cancer types, as predicted via the bioinformatics tool AcPEP, at https://app.cbbio.online/acpep/home (accessed on 14 March 2023). OAD means out of applicability domain. Concentrations below 50 µM indicate high anticancer activity.

Piscidin Peptide	Breast	Cervix	Colon	Lung	Prostate	Skin
Pleurocidin-like WF4	34.1 µM	10.7 µM	28.2 µM	71.8 µM	97.1 µM	7.0 µM
Pleurocidin	OAD	OAD	OAD	OAD	OAD	OAD
Pteroicidin-Alpha	7.8 µM	14.5 µM	26.7 µM	13.5 µM	28.0 µM	4.5 µM
Piscidin-3	33.2 µM	25.0 µM	62.4 µM	58.4 µM	226.7 µM	6.5 µM
Moronecidin *M.s*	15.7 µM	12.5 µM	23.9 µM	25.0 µM	82.6 µM	6.0 µM
Dicentracin	15.4 µM	12.7 µM	25.5 µM	26.3 µM	63.9 µM	6.8 µM
Moronecidin *M.c*	15.6 µM	14.4 µM	22.5 µM	25.1 µM	59.6 µM	8.0 µM
Pleurocidin-like WF3	6.9 µM	63.5 µM	116.1 µM	20.4 µM	100.5 µM	8.2 µM
Chrysophsin-3	9.6 µM	95.0 µM	62.4 µM	19.8 µM	19.3 µM	7.3 µM
Chrysophsin-2	OAD	OAD	OAD	OAD	OAD	OAD
Chrysophsin-1	OAD	OAD	OAD	OAD	OAD	OAD

## Data Availability

Not applicable.

## Supplementary Materials

The following supporting information can be downloaded at: https://www.mdpi.com/article/10.3390/antibiotics12050855/s1. Table S1: Pisc-Seq-counts.
